# Mid-term symptomatic relief after platelet-rich plasma infiltration in vulvar lichen sclerosus

**DOI:** 10.1007/s00403-023-02529-1

**Published:** 2023-01-19

**Authors:** Carola Medina Garrido, Alejandra Cano García, Lidia de la Cruz Cea, Ana B. Oreja Cuesta

**Affiliations:** 1 Universidad Alfonso X el Sabio, Campus de Villanueva de la Cañada, Madrid, Spain; 2 Department of Obstetrics and Gynecology, Section of Vulvar Diseases, Hospital Universitario del Tajo, Av. Amazonas Central s/n, 28300 Aranjuez, Madrid Spain

**Keywords:** Vulvar, Lichen sclerosus, PRP, Platelet-rich plasma, Infiltration, Symptom score, Grow curve model

## Abstract

**Purpose:**

Vulvar lichen sclerosus (LS) is a chronic, progressive, autoimmune dermatologic condition that causes cutaneous changes accompanied by pruritus and pain. There remains a small population with vulvar LS refractory to topical corticosteroids. Injection of platelet-rich plasma (PRP) has been reported to have positive effects on tissue repair. The aim of this pilot study was to evaluate changes in symptom scores during and after PRP vulvar infiltration.

**Methods:**

Three PRP infiltrations were administered to 28 female postmenopausal patients with biopsy-proved LS with unsatisfactory response to steroid therapy. Change in score according to the Clinical Scoring System for Vulvar Lichen Sclerosus (CSS) was measured on six occasions over the course of a year. We used growth curve modeling to measure change over the period of the study.

**Results:**

Women in our study experienced a statistically significant improvement in auto-assessed symptoms of vulvar lichen sclerosus, and this improvement appears to be maintained throughout the monitoring year.

**Conclusion:**

Platelet-rich plasma may have a role in symptom relief in certain cases of patients with LS that do not respond to first-line therapy.

**Supplementary Information:**

The online version contains supplementary material available at 10.1007/s00403-023-02529-1.

## Introduction

Lichen sclerosus (LS) is a chronic, progressive, autoimmune dermatologic condition, which predominantly affects the anogenital region. It is one of the most common dermatoses of the vulva in women in their 50 s and beyond, with great impact on quality of life [1, 2].

The vulvar skin appears thinned, fragile, and presents white sclerotic plaques, fissures and other dermal changes often accompanied by pruritus and burning. Lichen sclerosus also causes significant distortion of the vulvar anatomy, resulting in scarring and stenosis of the introitus. Dyspareunia is very common, with sexual dysfunction and symptoms such as soreness difficult to control.

Topical corticosteroids are the gold standard treatment, and they are both effective and safe. However, they require regular application, and treatment failure or intolerance can occur in clinical practice [3–5]. For patients who do not show improvement with this treatment, several other options may be tried: calcineurin inhibitors, topical and oral retinoids, steroid injections, ciclosporin, and methotrexate. The level of evidence is low for most of these agents, with the exception of the calcineurin inhibitors, which are considered second-line treatments for vulvar LS [6, 7].

Platelet-rich plasma is an autologous solution of highly concentrated platelets. Various growth factors and cytokines are released after the degranulation of platelets, which induce cellular proliferation, migration, differentiation, and extracellular matrix synthesis. Infiltration of this solution has been shown to have positive effects on tissue repair and wound healing, with minimal risk of adverse events [8, 9]. Owing to its regenerative effects and anti-inflammatory potential, PRP represents a novel approach for patients with LS, particularly in cases poorly responsive to topical therapies.

Existing research on this treatment is scarce and inconclusive: Goldstein initially presented in 2017 a pilot study in which 15 patients showed a decrease in histopathologic inflammation measured by two blinded dermatopathologists [10]. Later, Tedesco et al. published a study with 31 patients, showing clinical improvement after PRP infusion [11]. In 2019, the only randomized placebo-controlled trial comprising 30 patients treated with PRP, failed to show improvement, suggesting that autologous PRP does not adequately treat vulvar LS [12]. In the study with the largest number of patients to date (94 patients), both female and male patients had a significant reduction in symptoms after six months of PRP treatment, and showed improvement in sexual function and quality of life [13].

In this contradictory context, in which use of PRP therapy has grown significantly in recent years in multiple medical specialties, and most studies finding PRP to be favorable over control treatment [14], many women with vulvar lichen go years experiencing symptoms and plateau even with adequate treatment. In this work, we attempt to gather evidence of the evolution of women with LS during and after PRP infiltration.

## Methods

### Participants

Patients intolerant or unresponsive to topical steroid treatment or with poor symptom control were offered to participate in the study. There were no exclusion criteria except those related to comorbidities that could limit the ability of the patient to participate in the study. Patients were not permitted to use additional medications throughout the duration of the study.

A total of 28 female postmenopausal patients (mean age 66.6 years) with biopsy-proven LS were recruited. Written informed consent was obtained from all subjects or a legal surrogate. The patients were proposed to receive three sessions with PRP. One-year follow-up results are presented below. Five participants withdrew before completing treatment and 23 completed the study. Drop-outs are included in the statistical model. Any proprietary sampling contact information was approved by its owner.

### Procedure

Three PRP infiltrations were administered four to six weeks apart to affected areas 30 minutes after applying a Lidocaine 2.5% and Prilocaine 2.5% cream. A commercial device for preparing autologous PRP was used: a nurse collected 18 mL of blood by venipuncture filling one 20-mL syringe containing 2 mL of ACD-A. The blood was transferred into the *Hy-tissue 20 PRP* device (Fidia, Abano Terme, Italy) before centrifugation using the Omnigrafter 2.0 (Fidia, Abano Terme, Italy). Plasma was recovered using a 10-mL syringe through the Push-out system.

We injected about four mL of the collected PRP into the most affected areas of the vulva and perineum (areas where skin color and texture were not normalized such as white sclerotic plaques, fissures and erosions). The injections were made using a 27G caliber needle, and tangential injection 60° into the dermis.

### Instruments

For the purposes of this pilot study, data collected on six separate occasions were used (at baseline, one month after each infiltration, and at 6 and 12 months after the PRP treatment). The primary endpoint was change in score according to the Clinical Scoring System for Vulvar Lichen Sclerosus (CSS). CSS is a validated numerical rating scale that assesses the patient´s impression of the severity of the LS [15]. At home, each patient fills in a 1–10 score of symptoms (pruritus, burning, soreness and dyspareunia) at the beginning of the study (basal), one month after each session, in a six-month follow-up visit, and in a one-year follow-up visit. Each item is scored on a numeric scale ranging from 1 (no complaints) to 10 (severe complaints).

To track symptom response, patients were interviewed and examined after each treatment. Subjective improvement was noted by all patients (3-point scale: no improvement, some improvement, great improvement). Patients were also asked if they had had sexual relations in the last month.

### Statistical analysis

We estimated descriptive statistics for our study variables along the different time points: basal, after first infiltration, after second infiltration, after third infiltration, six months follow-up, and one year follow-up. Next, we used a model that allowed us to measure change over time of several evaluations that do not need to be equally spaced: growth curve model [16]. Specifically, we aimed to explore the trend of our study variables: subjective improvement, itchiness, burning and soreness. These trends might be flat, downward, or upward [17].

Growth curve, in contrast to ANOVA, allows the estimation of parameters using Full Information Maximum Likelihood (FIML). FIML does not impute any data, but rather uses each case's available data to compute maximum likelihood estimates. Even when data are missing, not at random, FIML can retrieve bias [18].

## Results

Descriptive data for the six repeated observations of vulvar symptoms are summarized in Table 1.

**Table 1:** data for the six repeated observations of vulvar symptoms

Variable	Time point	Mean	SD	MIN	MAX
Itchiness	Basal	6.8	2.3	1	10
	First infiltration	5.6	2.7	1	10
	Second infiltration	4.2	2.6	1	10
	Third Infiltration	3.9	3.1	1	10
	Six-month follow-up	4.5	2.5	1	9
	One-year follow-up	4.0	2.5	1	9
Burning	Basal	5.8	3.2	1	10
	First infiltration	5.1	3.0	1	10
	Second infiltration	4.3	3.0	1	10
	Third Infiltration	3.2	2.8	1	10
	Six-month follow-up	4.6	2.9	1	10
	One-year follow-up	3.5	2.0	1	8
Soreness	Basal	4.1	3.2	1	10
	First infiltration	2.8	2.9	1	10
	Second infiltration	3.0	2.7	1	9
	Third Infiltration	2.5	2.5	1	9
	Six-month follow-up	2.5	2.4	1	8
	One-year follow-up	1.6	1.7	1	7
Subjective improvement	First infiltration	1.9	0.8	1	3
	Second infiltration	2.3	0.9	1	3
	Third Infiltration	2.5	0.7	1	3
	Six-month follow-up	2.5	0.6	1	3
	One-year follow-up	2.6	0.6	1	3

Scores on itchiness, burning, soreness and subjective improvement according to the CSS over time

Long-lasting vulvar itching is the most frequent symptom in adults, and it is also the item with the highest baseline rating in our patients (nearly 7 points). After two infiltrations, patients show a significant decrease, reporting an average of 4.3 points, a value that is maintained on successive visits for up to one year. Burning is the second most important complaint. Our patients began the treatment with average values of around 6 and finished with a drop of 2 points on our scale. Pain or feeling sore is not the main symptom reported, yet a decrease in this is also observed after PRP infiltration.

When asked about improvement after each session of treatment, a positive evolution is observed. Patients reported an average of 2 (some improvement) after the first PRP infiltration and this progressed during the treatment towards a 3 (great improvement). It is noteworthy that the subjective feeling of improvement is maintained at follow-up, both at six-months and one-year visits.

Regarding sexual relations, 20 of the 28 patients recruited reported no sexual relations (71%). Given the small number of patients having sexual intercourse, evolution of dyspareunia was not considered in this study.

Patients reported mild to moderate pain after the procedure; however, no adverse outcomes (e.g., infection, bleeding) were reported.

### Growth curve models

In these analyses, we were interested in describing change over time in vulvar symptoms in our patients treated with PRP infiltration. Longitudinal data of the measured variables were used to construct the growth models for each item described below. Each line of the graphs corresponds to one of the 28 patients included, to avoid saturation the software displays the most representative cases. Bold line represents the mean.

**Fig. 1:**
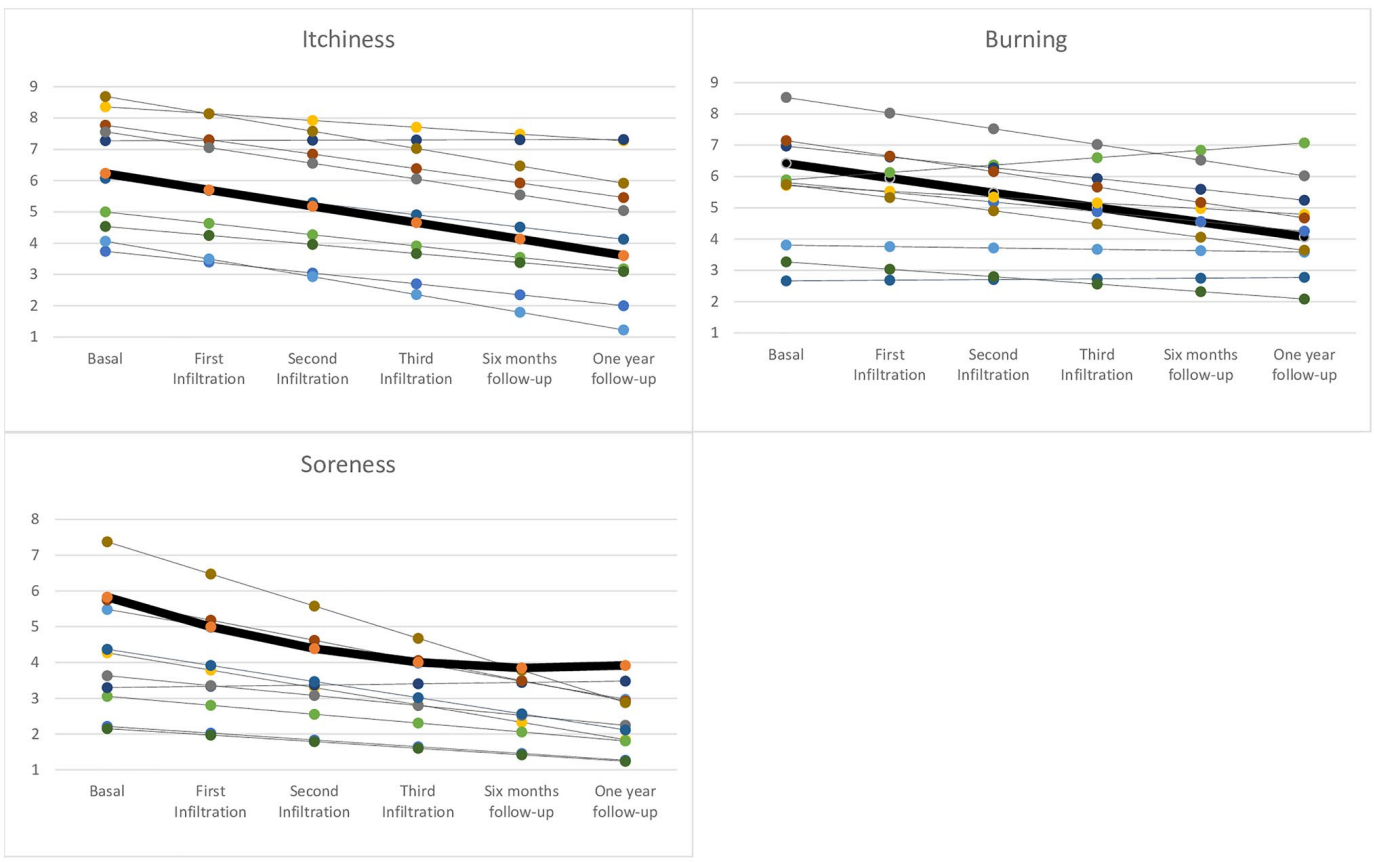
Estimated trends by the growth model in the score given by patients for the item “itching, burning and soreness” after each PRP infiltration and at follow-up visits. Each line of the graphs corresponds to one of the 28 patients included, to avoid saturation the software displays the most representative cases.

Figure 1 shows the evolution of the model estimated with our data, in which a downward trend is observed in each item. In the first variable, itching, the model shows a decrease at each time point of 0.5 with a *p* < 0.001. Burning decreases 0.312 at each time point with a *p* < 0.05. There was also a statistically significant downward trend for soreness (*p* < 0.001), with an average decrease of 0.348 per time point over the study period.

**Fig. 2:**
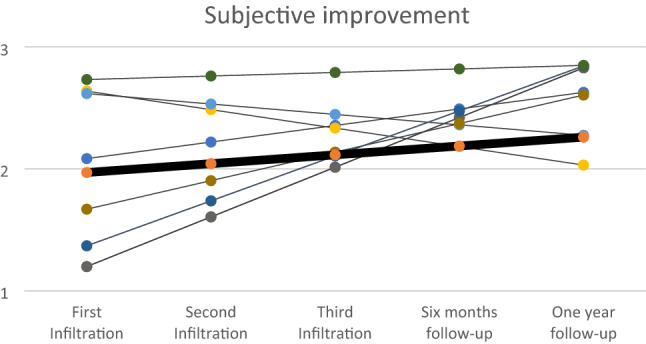
Estimated trends by the growth model in the score given by patients for the item “improvement” after each PRP infiltration and at follow-up visits.

Subjective improvement was also noted by all patients in a 3-point scale. When we estimate the trend with the growth model, the upward trend is maintained with an increase of 0.136 in every time point and a *p* < 0.05. (Fig. 2)

## Discussion

A definitive cure for LS does not exist, and there remains a group of patients who do not respond to steroid treatment. This failure in response is in many cases due to compliance problems or poor treatment tolerance [3, 4]. Other patients, despite major improvements in symptoms and signs, may have residual disease, with persistent negative effects on their well-being and quality of life [5]. The ideal treatment should aim at inducing relief of symptoms and preventing further anatomical changes and malignant transformation. Women in our study experienced a statistically significant improvement in all auto-assessed symptoms of vulvar lichen sclerosus, and this improvement appears to be maintained throughout the monitoring year.

Our findings regarding symptomatic relief are in line with other previous works [10, 11, 13], but most of the previous studies (except for the Goldstein [12]) present limitations such as the absence of a control group, as does this study.

Since uncontrolled trials are easier to implement than controlled trials, it must be recognized that this type of design cannot provide definite information regarding such hard clinical endpoints as effectiveness. In light of the limited experiences of the use of PRP to treat gynecological disorders, these first approximations are only the prelude to more robust, controlled trials.

Our study just focused on evaluating symptomatic improvement. Despite the importance of evaluating objective signs in clinical trials, it is very difficult to agree on any signs, architectural changes, or an overall global impression to assess vulvar LS disease severity, as was shown by Sheinis et al. [19]. Their results demonstrate a complete lack of consensus regarding perception of severity for signs and for disease severity among global experts. When patients are asked about assessing disease severity, they report irritation, fusion of the labia, soreness, itch, and decrease in quality of life [20]. Ultimately, parameters related to the symptoms of the disease. We did not use histopathological change as an outcome measure in this work because it requires an invasive procedure and because it is not a clinical outcome relevant to the well‐being of affected people.

It should be noted that, in the case of itching and burning, the curves of the model show an additive effect over time. This effect has also been observed in the intra-articular injection of PRP, as presented in the work of Filardo et al. [21]. This meta-analysis shows that the benefit increases over time, being not significant at earlier follow-ups but becoming clinically significant after 6 to 12 months. To our knowledge, there are no previous data on the application of PRP for vulvar lichen with a one-year follow-up.

Of concern is the association between VLS and subsequent vulvar squamous cell carcinoma. Findings have indicated that women compliant with topical corticosteroid treatment demonstrate lower rates of vulvar SCC compared to women who were inconsistent with this treatment [3]. This issue should always be discussed with patients before offering new therapies such as PRP infiltrations.

What we provide with this work is a medium-term view of the changes in symptoms in women with LS during and after PRP infiltrations. The symptomatic relief is maintained during the follow-up period, which reaches one year. Furthermore, the treatment of data with growth curve modelling allows an adequate management of dropouts and missing data, improving the external validity of the work.

Future directions for LS management should focus on objective and measurable post-treatment improvement from double-blind controlled studies, with standardized PRP processing and application, to improve recommendations in adjuvant treatment for LS.

The rise in such regenerative therapies warrants further studies to standardize cellular clinical adjuvants in inflammatory skin conditions such as LS.

## Conclusions

Platelet-rich plasma infiltrations may have a role in symptom relief in select cases of patients with severe LS that do not respond to first-line therapy, or where other therapies are poorly tolerated or contraindicated.

## Supplementary Information

Below is the link to the electronic supplementary material.

Supplementary file1 (JPEG 285 kb)

Supplementary file2 (JPEG 304 kb)

Supplementary file3 (JPEG 279 kb)

## Data Availability

The datasets generated during and/or analysed during the current study are available from the corresponding author on reasonable request.

## Abbreviations

ACD-A: Anticoagulant citrate dextrose solution, Solution A
ANOVA: Analysis of variance
CSS: Clinical scoring system for vulvar lichen sclerosus
FIML: Full information maximum likelihood
LS: Lichen sclerosus
PRP: Platelet-rich plasma
SD: Standard deviation
SCC: Squamous cell carcinoma
