# Supplementary figures and images for: Mid-term symptomatic relief after platelet-rich plasma infiltration in vulvar lichen sclerosus
Source: Arch Dermatol Res. 2023 Jan 19;315(6):1527–32. doi: 10.1007/s00403-023-02529-1 (PMC10338614; doi:10.1007/s00403-023-02529-1)

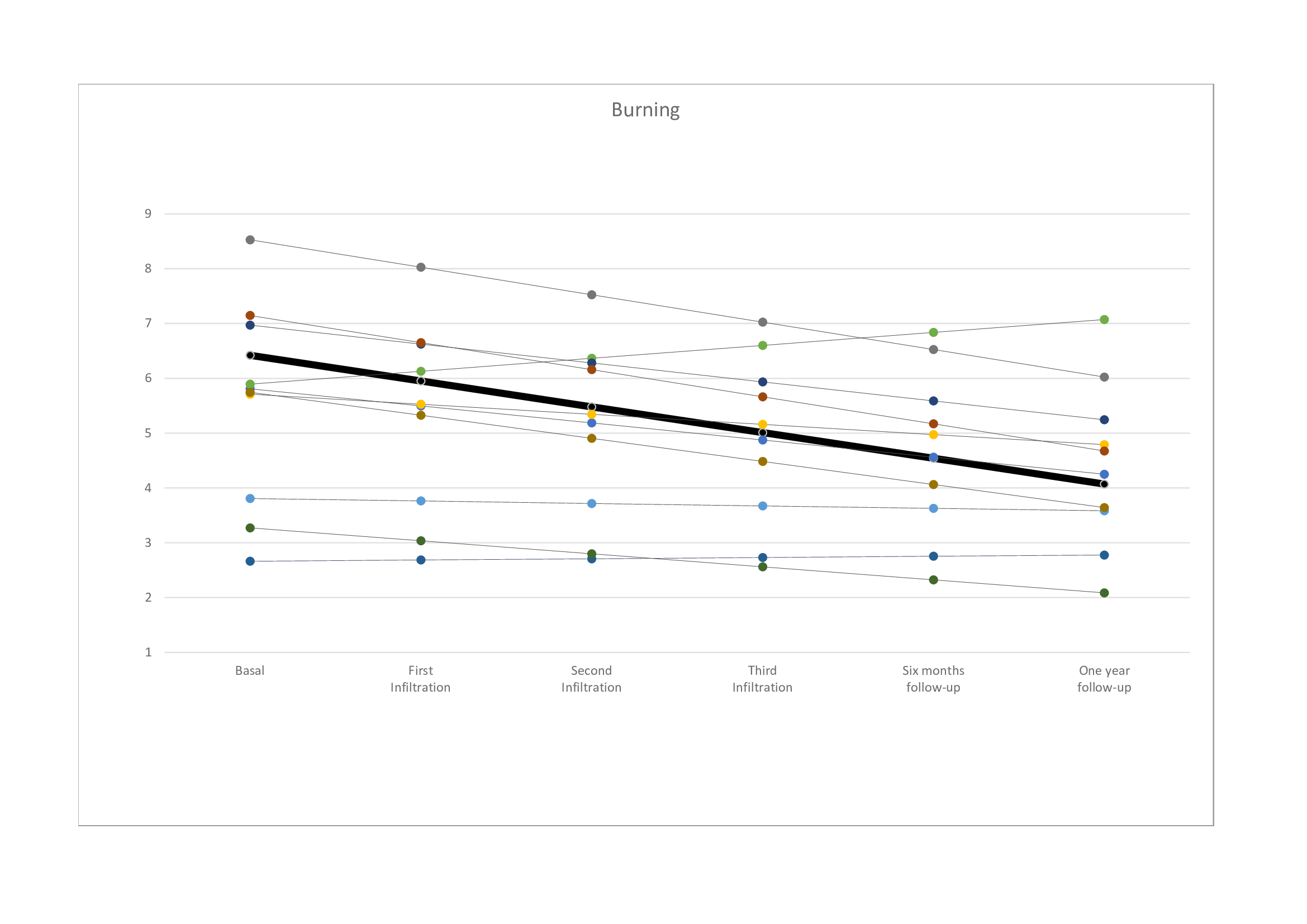

Supplement: Supplementary file 1 — Supplementary file1 (JPEG 285 kb) [file 403_2023_2529_MOESM1_ESM.jpeg]

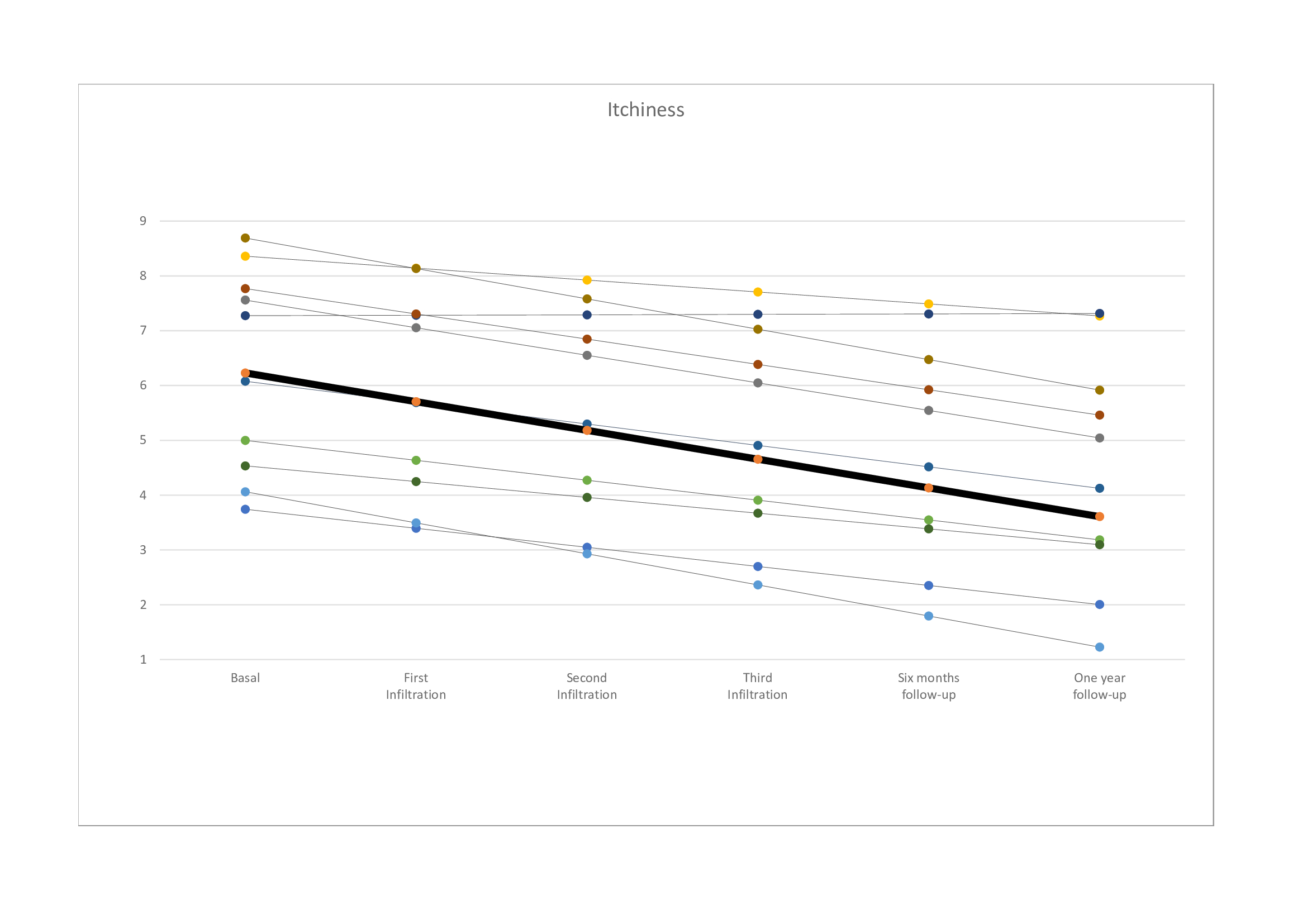

Supplement: Supplementary file 2 — Supplementary file2 (JPEG 304 kb) [file 403_2023_2529_MOESM2_ESM.jpeg]

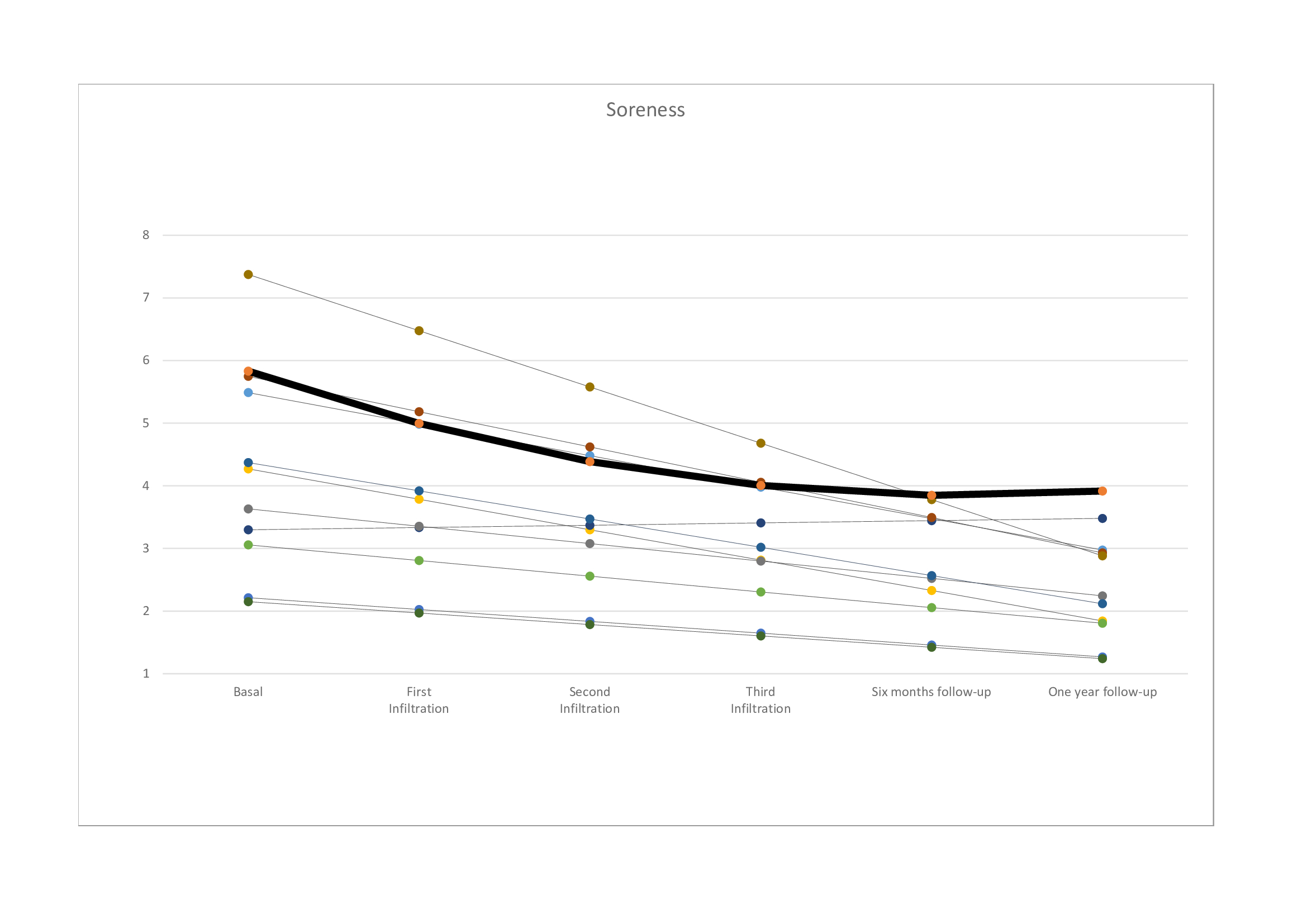

Supplement: Supplementary file 3 — Supplementary file3 (JPEG 279 kb) [file 403_2023_2529_MOESM3_ESM.jpeg]
